# Infarct burden following multivessel PCI vs. infarct-only PCI in patients with acute STEMI: the Glasgow PRAMI CMR sub-study

**DOI:** 10.1186/1532-429X-17-S1-O9

**Published:** 2015-02-03

**Authors:** Kenneth Mangion, David Carrick, Alexander R Payne, John D McClure, Maureen Mason, Mark Petrie, Margaret McEntegart, Hany Eteiba, Keith G Oldroyd, Colin Berry

**Affiliations:** 1 BHF Glasgow Cardiovascular Research Centre, University of Glasgow, Glasgow, UK; 2 Golden Jubilee National Hospital, Clydebank, UK

## Background

In the Preventive Angioplasty in Myocardial Infarction trial (PRAMI; ISRCTN73028481), immediate multivessel PCI (MV-PCI) of non-IRA (infarct related artery) lesions in patients with acute ST elevation myocardial infarction (STEMI) and multivessel coronary disease (MVD) improved long term prognosis. We assessed infarct distribution and size in a pre-specified cardiac magnetic resonance (CMR) sub-study.

## Methods

In this single centre prospective sub-study, PRAMI participants were invited to undergo 1.5 Tesla CMR 1 week and 1 year after primary PCI. The CMR scans were analysed using semi-automated software by a clinician blinded to treatment group assignment and clinical outcomes. The presence and extent of infarction were assessed quantitatively with late gadolinium enhancement (LGE) imaging (Gadovist, 0.1 mmol/kg). The infarct was delineated as an area of myocardial enhancement (cm^2^) using a signal intensity threshold of >5SDs above a remote region, and expressed as a % of total LV mass. The incidence of new LGE in non-infarct related artery territories at baseline and 1 year were assessed. Data were analysed by an independent statistician.

## Results

Of 465 randomised trial participants in 6 UK hospitals, 138 (30%) were enrolled in Glasgow. Of these 80 patients underwent CMR 1 week post primary PCI of whom 41 (51%) were in the multi-vessel PCI group and 39 (49%) were in the IRA-only group. At 1 year, 69 (86%) patients had a follow up CMR scan. Infarct size and distribution are described in Table 1.

**Table 1:** Infarct size and distribution in non-infarct artery territory in the randomised PRAMI trial participants (n=80) in Glasgow.

	Infarct-only PCI n = 39 (49%)	Multivessel PCI n=41 (51%)	p
1 week post-MI			

Infarct size, % LV′	16.9 (14.0)	13.9 (12.1)	0.32

Infarct in non-IRA territory, n (%)	0 (0)	0 (0)	1.00

1 year post-MI			

Infarct size, % LV	13.9 (10.1)	11.1 (11.2)	0.20

Change in infarct size from baseline, % LV mass*	-2.23 ( -9.97, 0.56)	-1.73 (-7.10, 0.94)	0.60

Infarct in non-IRA territory, n (%)	2 (5.1)	3 (7.3)	1.00

′ mean (standard deviation)

*median (interquartile range)

## Conclusions

Infarct size and distribution were similar in patients treated by MV-PCI or IRA-only PCI. MV-PCI is not associated with additional MI acutely which supports the safety of this procedure in line with the benefits observed with preventive PCI in PRAMI.

## Funding

Golden Jubilee National Hospital; PRAMI was funded by Barts and the London Charity.

